# The Ups and Downs of Metabolism during the Lifespan of a T Cell

**DOI:** 10.3390/ijms21217972

**Published:** 2020-10-27

**Authors:** Renu Balyan, Namrata Gautam, Nicholas R.J. Gascoigne

**Affiliations:** Immunology Programme and Department of Microbiology and Immunology, Yong Loo Lin School of Medicine, National University of Singapore, 5 Science Drive 2, Singapore 117545, Singapore; renubalyan89@gmail.com (R.B.); e0012553@u.nus.edu (N.G.)

**Keywords:** immunometabolism, T cell, aerobic glycolysis, fatty acid oxidation, T cell activation, CD4 T cell differentiation, metabolic reprogramming

## Abstract

Understanding the various mechanisms that govern the development, activation, differentiation, and functions of T cells is crucial as it could provide opportunities for therapeutic interventions to disrupt immune pathogenesis. Immunometabolism is one such area that has garnered significant interest in the recent past as it has become apparent that cellular metabolism is highly dynamic and has a tremendous impact on the ability of T cells to grow, activate, and differentiate. In each phase of the lifespan of a T-cell, cellular metabolism has to be tailored to match the specific functional requirements of that phase. Resting T cells rely on energy-efficient oxidative metabolism but rapidly shift to a highly glycolytic metabolism upon activation in order to meet the bioenergetically demanding process of growth and proliferation. However, upon antigen clearance, T cells return to a more quiescent oxidative metabolism to support T cell memory generation. In addition, each helper T cell subset engages distinct metabolic pathways to support their functional needs. In this review, we provide an overview of the metabolic changes that occur during the lifespan of a T cell and discuss several important studies that provide insights into the regulation of the metabolic landscape of T cells and how they impact T cell development and function.

## 1. Role of Cellular Metabolism in Regulating Thymic Development

T cell development is a tightly regulated process and involves multiple proliferation and selection steps in the thymus. Firstly, double-negative (CD4−CD8−: DN) thymocytes initiate V(D)J rearrangement of the T cell receptor (TCR) β chain locus. Once it is successfully rearranged, the DN cells undergo β-selection and transition through the DN3 and DN4 stages. These cells undergo rapid growth and proliferation, supported by an induction of the glucose transporter Glut1 and increased glycolysis [1,2]. However, Glut1 is downregulated as the cells mature to more quiescent CD4^+^CD8^+^ double-positive (DP) and then CD4^+^CD8^−^ and CD4^−^CD8^+^ single-positive (SP) cells. A number of metabolic pathways orchestrate changes to meet the bioenergetic demands required at each step of thymocyte development [3]. Here, we provide an overview of the metabolic regulators and pathways governing thymocyte development.

Notch signaling: Notch signaling plays a significant role in early thymocyte development. Mice with an inducible knockout of Notch1 showed a developmental block of immature thymocytes at the CD25^−^CD44^+^ DN1 stage [4], whereas the expression of constitutively active Notch1 in hematopoietic stem cells (HSC) blocks B cell differentiation and leads to ectopic development of immature DP T cells in the bone marrow [5]. Apart from HSC differentiation towards the T cell lineage, Notch signaling is also important for β-selection of DN thymocytes [6]. DN thymocytes atrophied when deprived of Notch signaling, with lower Glut1 expression and a decreased glycolytic rate, eventually leading to apoptotic death. Absence of Notch signaling in DN thymocytes leads to lowered Glut1 expression and glycolytic rate, thereby causing apoptosis [6].

PI3K/AKT pathway: Notch signaling promotes glucose metabolism in thymocytes by activating the phosphatidylinositol-3 kinase (PI3K)-protein kinase B, also known as the AKT signaling pathway. Inhibition of PI3K or AKT in DN thymocytes has been shown to suppress glucose metabolism. Overexpression of constitutively active AKT1 restores glucose metabolism in Notch-deprived thymocytes and rescues the early pre-T-cell development block caused by impaired Notch signaling [6]. Mice with impaired PI3K signaling due to deletion of PDK1 or both PI3Kδ and γ isoforms had a developmental block at the DN3/DN4 stage of thymocyte development [7,8,9]. Akt1-/- Akt2-/- thymocytes manifest a severe developmental block at the DN3 stage, and Akt1-/- Akt2-/- DN3 cells show reduced glucose uptake and die in response to TCR stimulation in vitro. This developmental defect is due to apoptosis, which is partially caused by decreased cellular growth and metabolism at the DN3 stage. AKT has been shown to protect thymocytes from cell death during the β-selection step [10].

PI3K–AKT signaling is known to activate the mammalian target of rapamycin (mTOR), thereby promoting glucose metabolism required to support cell growth and proliferation. Impaired PI3K signaling blocks the transition of DP thymocytes to single-positive CD4 and CD8 T cells [11]. PTEN (phosphatase and tensin homologue) is a phosphatase that is well known as an important negative regulator of the PI3K pathway. Thymocytes from mice lacking the microRNA cluster miR-181a1b1 show a significant increase in PTEN expression. These cells show reduced glucose uptake and glycolytic rate, and these mice have a deficiency in the DP cell population and completely lack NKT cells [12]. These findings suggest the impact of the PI3K–AKT pathway on thymocyte development via its role in regulating thymocyte metabolism.

IL-7 cytokine: IL-7 is known to play an important role in the survival of naive T cells by increasing the expression of the anti-apoptotic factor B cell lymphoma 2 (Bcl-2) [13,14]. In addition to maintaining the survival of lymphocytes, IL-7 signaling promotes the growth and proliferation of DN4 cells. It does so by increasing the expression of transferrin receptor CD71 [15] and the amino acid transporter CD98 [16]. Mice deficient in IL-7 or IL-7Rα show defects in T cell development [17,18]. In addition to signaling via the JAK3– STAT5 pathway, IL-7 can also activate PI3K [19,20].

**Figure 1:**
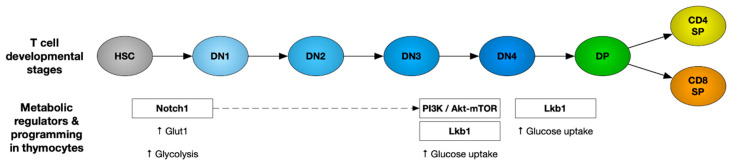
Metabolic regulators and pathways governing thymocyte development. Upregulation of glucose metabolism is the foremost requirement of developing thymocytes regulated by Notch, PI3K–AKT–mTOR and Lkb1 signaling pathways during different stages of T cell development as depicted here. HSC: hematopoietic stem cells, DN: CD4^−^/CD8^−^ double-negative thymocytes, DP: CD4^+^/CD8^+^ double-positive thymocytes, and SP: CD4^+^CD8^−^ or CD8^+^CD4^−^ single positive.

AMPK: The AMP-activated protein kinase (AMPK) is an important metabolic regulator that senses energy deficiency and maximizes ATP generation by promoting catabolic pathways and inhibiting anabolic processes that consume ATP [21]. It is activated by an increased ratio of AMP to ATP through phosphorylation by the tumor suppressor liver kinase B1 (Lkb1) and calcium/calmodulin-dependent protein kinase II (CaMKKII) [22,23]. The AMPK pathway is essential for the cellular metabolism of HSCs. Lkb1 deletion was reported to trigger HSC proliferation, followed by their premature depletion. Lkb1-deficient HSCs showed lower mitochondrial membrane potential and ATP levels [24]. Mice with T-cell-specific Lkb1 deletion displayed a block in thymocyte development. These mice had higher frequencies of DN thymocytes with accumulation in the DN3 stage and a reduction in DP and SP thymocyte populations [25,26,27]. However, AMPKα1 deletion did not mimic the defect in thymocyte development seen in LKb1-deficient mice [28], suggesting that there may be additional substrates of Lkb1 involved. The various metabolic regulators that play an important role in thymocyte development have been represented in Figure 1.

TORC1 and αβ versus γδ T cell fate determination: During thymocyte development, differentiation into αβ T cells versus γδ T cells happens at the DN3 stage. Signaling via Notch1, IL-7, and the pre-TCR are required for the transcriptional and metabolic changes essential for differentiation [6]. This metabolic reprogramming is primarily dependent on the mammalian target of the rapamycin C1 (mTORC1) complex. mTOR signaling is transmitted via two complexes, mTORC1 containing a scaffold protein RAPTOR and mTORC2 containing a scaffold protein RICTOR [29]. A recent study showed that mTORC1 couples microenvironmental cues with metabolic pathways to orchestrate the reciprocal development of two fundamentally distinct T cell lineages, αβ T cells and γδ T cells [30]. Developing thymocytes depend on glycolysis, oxidative phosphorylation (OXPHOS), and mTORC1 signaling. Loss of regulatory-associated protein of TOR (RAPTOR)-mediated mTORC1 activity has been shown to impair the development of αβ T cells while promoting the development of γδ T cells [30]. This has been shown to be associated with disrupted metabolic remodeling of oxidative and glycolytic metabolism. Thus, mTORC1-dependent metabolic signaling has been established as a decisive factor for αβ T cell versus γδ T cell fate determination.

## 2. Metabolic Reprogramming during T Cell Activation

T cells are a prime example of how cellular metabolism is dramatically modified to support the specific requirements and functions of each cell state. T cell activation requires metabolic reprogramming so that activated T cells have the appropriate nutrients to meet their functional demands. Activated T cells need to grow rapidly, proliferate, and carry out their effector function. As a result, the cellular metabolism is modified in order to produce the biosynthetic precursors required for the bioenergetically demanding processes of cell growth and proliferation. Resting naive T cells rely on oxidative metabolism to efficiently produce energy. They take up glucose at a very low rate in order to supply the energy required to maintain housekeeping functions [31]. Naive T cells skew towards OXPHOS, whereas activated T cells skew towards aerobic glycolysis. This metabolic shift in T cells from OXPHOS to aerobic glycolysis is the activation benchmark known as the Warburg effect [32]. After antigen clearance, the cells that survive to become memory T cells return to a more quiescent oxidative metabolism [31].

Upon activation, T cells meet the demand of maintaining sufficient metabolites for cell growth and proliferation by increasing glucose and glutamine metabolism. Studies on peripheral blood leukocytes showed that aerobic glycolysis and lactate production are dramatically increased upon stimulation [32]. Mitogen-activated thymocytes [33] and peripheral T cells [34] also show similar metabolic changes. A rapid increase in Glut1 expression, indicating an increase in glucose uptake, has been reported during acute in vivo T cell stimulation [35]. This induction of glucose uptake is essential for proper T cell function. Transgenic expression of Glut1 in T cells leads to excessively increased glucose uptake, resulting in increased cytokine production and proliferation and ultimately causing lympho-proliferative disease [35,36]. Conversely, nutrient insufficiency or direct metabolic inhibition has been reported to prevent T cell activation and proliferation [36] and, in some cases, leads to anergy or cell death [37]. Metabolic competence has been shown to correlate with the ability of T cells to respond to TCR-mediated stimulation [38]. Variation in the glucose uptake capacity of naive T cells can regulate T cell activation and differentiation outcomes [38,39]. Thus, metabolic reprogramming is intricately involved with T cell growth and function.

Glutamine is another critical substrate used by T cells during activation. Depletion of glutamine in the culture media has been found to impair T cell proliferation [40,41]. Glutamine is metabolized by glutaminolysis, and the intermediates produced during this process enter the Tricarboxylic acid cycle (TCA) cycle. Glutamine also acts as a nitrogen donor for the synthesis of purine and pyrimidines and is therefore essential for synthesis of nucleotides required during proliferation [42].

In addition to the enhancement of glucose and glutamine uptake, T cell activation also leads to increased rates of protein synthesis [43]. Initiation of protein synthesis is regulated by mTOR, an evolutionarily conserved checkpoint protein kinase, which senses nutrient availability and metabolic stress to control growth and proliferation. mTOR can be regulated in two ways, first being the conformational changes in mTOR–RAPTOR complex to enhance accessibility to mTOR substrates, in the presence of nutrients [44]. Secondly, various growth factors can also regulate mTOR activity by its upstream regulators such as PI3K (phosphatidylinositol-3 kinase), PTEN, and TSC1/TSC2 (tuberous sclerosis complex) [45]. TCR and costimulatory signaling activates PI3K, which further activates AKT. Co-ligation of TCR and CD28 maximally activates AKT, which induces glycolysis and phosphorylates mTOR. Thus, increase in glucose transporter expression, glucose uptake, and glycolysis, as well as enhanced protein synthesis in response to TCR-mediated stimulation, are dependent on PI3K activity. Inhibition of mTOR by rapamycin leads to cell cycle arrest and is a potent T cell immunosuppressant [46]. The T-cell-specific protein Themis, which is important in thymocyte positive selection, has recently been shown to affect the AKT pathway and its downstream effects in mature T cells [47], and also affects metabolism [48,49].

mTOR regulates protein synthesis via multiple phosphorylation targets, including p70S6 kinase, a regulator of ribosome function, and 4E-BP1, an inhibitor of translation [50]. Mitogens are known to induce the activation of pS6 kinase, subsequently leading to phosphorylation of the S6 ribosomal protein, which correlates with an increase in translation of particular mRNA transcripts that encode proteins involved in cell cycle progression, other ribosomal proteins, and elongation factors necessary for translation [51]. mTOR also directs the cell surface expression of multiple nutrient transporters, including amino acid transporters and low-density lipoprotein receptor, in response to PI3K/AKT signaling [52]. Thus, control of glucose transport as well as protein synthesis is coordinated with import of other nutrients required for cell growth via the PI3K/AKT/mTOR signaling axis.

The transcription factor c-myc regulates many genes essential for cell cycle as well as cellular metabolism. c-myc controls glycolysis by promoting the expression of multiple glycolytic genes including Glut1, lactate dehydrogenase A, and hexokinase 2 [53], as well as promotes glutaminolysis by increasing the expression of glutaminase [54]. In T cells, c-myc expression is induced upon activation and promotes cell growth and entry into the cell cycle [55]. Genetic deletion of c-myc in T cells inhibits the upregulation of glycolytic and glutaminolytic gene expression in activated T cells, leading to a failure of c-myc-deficient cells to proliferate. Wang et al. suggested a pivotal role for c-myc in T cell metabolism using an in silico approach, demonstrating that multiple gene targets of c-myc correlate with changes in metabolic gene expression upon T cell activation [55].

The shift in the energetic equilibrium towards the glycolytic pathway in effector T cells is accompanied by several molecular and morphological changes. One such indispensable change is mitochondrial morphology and mass [56]. To efficiently carry out glycolysis over OXPHOS, mitochondrial scissors, i.e., a proteolytic enzyme, the GTPase dynamin-related protein 1 (Drp1), recruits a complex of proteins to chop up the mitochondria leading to a process termed mitochondrial fission. The effector cell mitochondrial morphology is described as “punctate”, referring to the dot-like appearances of the fissed mitochondria. During fission, mitochondrial cristae are loosened, as a result of which the enzymes of the electron transport chain (ETC) become sparsely placed, reducing the efficiency of electron transport, thus favoring glycolysis [57].

Metabolism in memory T cells: After antigen clearance, the majority of effector T cells die by apoptosis. The small percentage of cells that survive to become memory T cells return to a more quiescent state as they do not to have the same bioenergetic demands as effector T cells. The memory cells have a better recall response and are therefore more protective following rechallenge with the antigen. The signaling and metabolic pathways that regulate the generation of memory cells are therefore of great interest.

**Figure 2:**
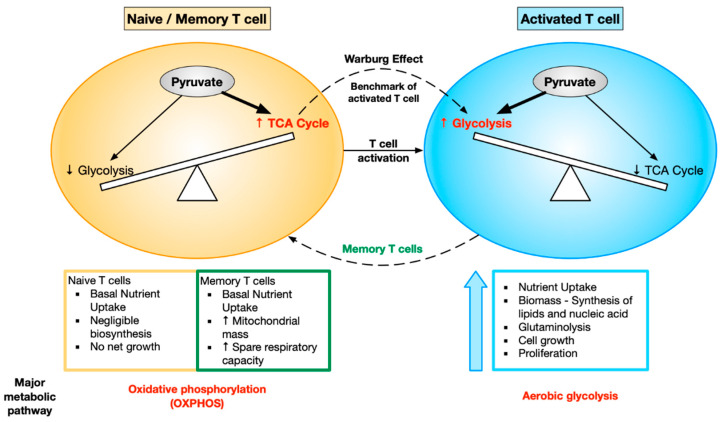
Different metabolic demands of naive, activated, and memory T cells. Naive and memory T cells skew towards oxidative phosphorylation (OXPHOS), whereas activated T cells skew towards aerobic glycolysis. This metabolic shift in the T cells (OXPHOS → aerobic glycolysis) is the activation benchmark known as the Warburg effect. The naive T cells are quiescent in nature, with no net growth, whereas activated T cells show boosted nutrient uptake and growth in biomass, ultimately leading to cell growth and proliferation. Memory T cells have basal nutrient uptake, but they show increased mitochondrial mass and spare respiratory capacity, helping them increase their longevity.

Tumor necrosis factor (TNF) receptor-associated factor 6 (TRAF6) is required for CD8 T cells to switch from glycolytic to oxidative metabolism. TRAF6-deficient T cells are impaired in their ability to form antigen-specific memory T cells due to defects in oxidative fatty acid metabolism (FAO) [58]. It was found that augmenting FAO in CD8 T cells by treating mice with metformin (AMPK activator) or rapamycin (mTOR inhibitor) promotes memory T cell generation in vivo [58,59]. Interestingly, these findings suggest that targeting FAO post-vaccination could enhance the development of CD8 memory T cells. In addition to relying on FAO, memory T cells maintain higher mitochondrial spare capacity than naive or effector T cells. IL15 has been shown to play an important role in the cellular metabolism of memory T cells by promoting mitochondrial biogenesis. It enhances the expression of carnitine palmitoyl transferase 1a (CPT1a), a mitochondrial enzyme crucial for FAO. Over-expression of CPT1A in T cells increases the survival and recall response of antigen-specific CD8 T cells in vivo [60]. From these studies and multiple others, it is clear that bioenergetic distinctions exist between effector and memory T cells. Unlike effector T cells, which primarily rely on glycolysis, memory T cells have a higher spare respiratory capacity and mitochondrial mass and predominantly rely on FAO. The changes in metabolic demands of T cells upon activation have been depicted in Figure 2.

## 3. Cellular Metabolism and CD4 T Cell Differentiation

The distinct functional requirements of specialized CD4 T cell subsets require specific metabolic programs in order to meet their differing energetic and biosynthetic demands. Here, we review the role of cellular metabolism in regulating CD4 T cell differentiation.

### 3.1. Metabolic Regulation of Th1 Cells

Th1 cells mainly secrete IFNγ, which allows these cells to eliminate intracellular pathogens such as viruses and some bacteria. It also activates macrophages and enhances their phagocytic activity. T-box transcription factor (T-bet), the master regulator for Th1 differentiation, not only upregulates a gene network to promote Th1 differentiation but also suppresses the genes specific to Th2 and Th17 development. *T-bet* expression is chiefly regulated by signal transducer and activator of transcription 1 (STAT1), which is IL-12 dependent [61,62].

Th1-polarised CD4 T cells adopt aerobic glycolysis as their preferred pathway for energy production and show increased surface expression of Glut1 receptors [35,63,64,65]. Glycolysis is not only important for these effector cells to increase their biomass but is also essential for production of IFNγ. It was shown that T cells, which do not primarily rely on glycolysis, do not engage GADPH, leaving it free to bind to the 3′ UTR of Ifng mRNA, conferring post-transcriptional control over IFNγ production [63]. However, engagement of GAPDH in aerobic glycolysis releases Ifng mRNA to be translated, leading to its efficient production. Another study proposed that to maintain aerobic glycolysis and further support Th1 differentiation, lactate dehydrogenase A (LDHA) confers epigenetic control over the Ifng locus, and hence its expression in these effector T cells is a major prerequisite. This is so because LDHA-deficient T cells had drastically reduced histone activation, H3K9 acetylation marks, at the Ifng locus [65]. Therefore, these cells could not produce IFNγ efficiently. This hypothesis was supported in vivo as well, when protection was conferred from a lethal Th1-mediated autoinflammatory disease upon deletion of LDHA in T cells only [65].

Along with glycolysis, Th1 cells also rely upon glutaminolysis, which is the breakdown of glutamine, for their proliferation and growth [64]. Despite Th1 polarizing conditions, CD4 T cells that lack a glumatine supply generate Foxp3+ T regulatory cells (Treg) [66]. This effect is rescued by addition of a cell permeable α-ketoglutarate analogue (dimethyl-2-oxoglutarate) [67]. Glutaminolysis leads to the production of α-ketoglutarate, which promotes Th1 differentiation by enhancing T-bet expression [67].

Apart from glutamine, other branched-chain amino acids like valine, leucine, and isoleucine, and aromatic amino acids like phenylalanine, tyrosine, and tryptophan, are required by CD4 T cells for their proliferation and in vitro differentiation into Th1 cells [68]. Therefore, the expression of amino acid transporter CD98, which is responsible for uptake of the branched and aromatic amino acids, is of utmost importance for in vitro Th1 differentiation [68].

mTORC1 is the principal metabolic regulator of Th1 CD4 T cells. It is regulated via the activation of the PI3K–AKT signaling cascade. Inhibition of mTORC1 activation by deletion of Ras Homologue Enriched in Brain (Rheb) (activator of the mTORC1 pathway) leads to the suppression of Th1 differentiation. mTORC1 phosphorylates T-bet, whereas its inhibition reduces T-bet-dependent IFN-γ production [69]. HIF-1α, a known downstream target of mTORC1, hampers Th1 effector functions opposing the pro-Th1 effects promoted by mTORC1 [70,71]. In addition, deletion of HIF-1α isoform I.1 in T cells enhances immunity in a model of bacterial infection [72]. Despite this, the mechanism by which HIF-1α actually regulates Th1 differentiation still remains to be explored.

### 3.2. Metabolic Regulation of Th2 Cells

Th2 cells are involved in combatting infections caused by extracellular parasites, inclusive of helminths. Th2 cells mainly secrete IL-4, IL-5, and IL-13. IL-4 mediates IgE class switching in B cells. It also upregulates low-affinity IgE receptor (Fc*ε*RI) on mononuclear phagocytes and B lymphocytes and high-affinity IgE receptor (Fc*ε*RII) on basophils and mast cells. It even initiates degranulation of these cells, releasing active metabolites like serotonin and histamine [73]. Since eosinophils are known to express high amounts of IL-5R on their surface, they are a major target of IL-5, resulting in their activation and upregulation of CD11b while inhibiting apoptosis [74]. Secretion of IL-4, IL-5, and IL-13 leads to IL-25 expression. This enhances Ig secretion, IgE switching, eosinophilia, and mucus production, therefore further aggravating the Th2 response.

IL-4-induced STAT6 is the major transcription factor required for Th2 differentiation as it upregulates the expression of GATA3 (GATA-binding protein), the master regulator of Th2 [75,76,77]. GATA-3 promotes Th2 differentiation by regulating three mechanisms: enhancement of Th2 cytokine production, Gfi-1-mediated selective proliferation of Th2, and suppression of Th1 differentiation by interacting with T-bet [78]. GATA3 also downregulates STAT4 to suppress Th1 differentiation [79].

Th2 CD4 T cells predominantly undergo aerobic glycolysis in order to proliferate and perform their effector functions, just like the Th1 cells [80]. In vitro assays performed on Th1, Th2, and Th17 CD4 T cells show that they are all glycolytic in nature. However, amongst these subsets, Th2 has been classified as the most glycolytic and shows the highest expression of Glut1 [64,81].

It is noteworthy that all the T helper lineages Th1, Th17, and Th2 are mTOR-dependent, and rapamycin inhibition of mTOR skews the differentiation pattern towards Tregs [80,82,83]. Furthermore, all three of these lineages require mTORC1 activation because deletion of its scaffolding protein, RAPTOR, inhibits their effector differentiation [84]. However, mTORC2 can be classified as an indispensable metabolic regulator required for the differentiation, function, and metabolism of Th2 CD4 T cells [85]. This is supported through the following observations. Th2 differentiation remains unaffected upon Rheb-dependent activation of mTORC1; however, Th1 and Th17 are hampered. On the other hand, Th2 differentiation is hampered upon T-cell-specific deletion of a scaffolding protein of the mTORC2 complex, rapamycin-insensitive companion of mammalian target of rapamycin (RICTOR), but Th1 and Th17 differentiation remain unaffected [85]. In addition to this, mTORC2 inhibits suppressor of cytokine signaling-5 (SOCS5), which blocks Th2 differentiation by suppressing IL-4-dependent STAT6 [86]. Moreover, activation of mTORC2 downstream targets, Ras homolog gene family member A (RhoA) and serum/glucocorticoid regulated kinase 1 (SGK1), plays an important role in Th2 differentiation. RhoA coordinates glycolysis to Th2 differentiation and allergic airway inflammation by regulating IL-4 receptor mRNA expression and Th2-specific signaling events [87]. However, RhoA signaling is not required for Th2 maintenance [88]. SGK1 promotes Th2 commitment while blocking differentiation into Th1 lineage and inhibits the degradation of JunB [89], a transcription factor that controls the Th2 cytokine program [90].

Interestingly, studies have pointed out that some additional metabolic programming occurs in Th2 cells entering inflamed tissue sites. Therefore, along with the requirements of aerobic glycolysis and mTORC2 signaling exclusive to Th2 cells residing in the lymph nodes, tissue-migrated Th2 cells display lipid metabolism as their prominent feature. For instance, a recent study revealed increased expression of genes involved in lipid metabolism in Th2 cells present in the airways of mice challenged with house dust mite antigens [81]. Assays for Transposase-Accessible Chromatin (ATAC) analysis showed an increase in chromatin accessibility at various gene loci involved with lipid metabolism in Th2 cells over the other T cell subsets in the lung or naive CD4 T cells in the lymph nodes [81]. Using key inhibitors of lipid metabolism, a drastic reduction was seen in Th2 pathologies such as goblet cell metaplasia, airway eosinophilia, Th2 cell expansion, and production of IL-13 and IL-5 [81].

Various studies have highlighted the importance of peroxisome proliferator activated receptor gamma (PPAR-γ), a nuclear receptor that is shown to regulate lipid metabolism in Th2 cells [91,92,93,94,95]. PPAR-γ expression is perhaps induced by IL-4R ligation and STAT6 activation in Th2 cells, as seen in macrophages and dendritic cells [96,97,98]. Reduction in Th2-mediated immunity towards the nematode *Heligmosomoides polygyrus* and improved pathologies in Th2-driven airway inflammation models were observed in absence of PPAR-γ [92,94]. This is thought to be because absence of PPAR-γ led to the loss of the ability to screen the ligands in the lung and also impaired the expression of IL-13 and IL-5 by Th2 cells [94]. On the other hand, no defects were pointed out in the initial activation of Th2 cells in lung-draining lymph nodes [94]. This indicates that presence of PPAR-γ is more critical for the functioning of tissue-migrated Th2 cells.

The substantial impact of PPAR-γ on Th2-mediated pathologies could be explained by its enriched binding sites at accessible chromatin regions and also at critical target genes such as Ap1, Ets1, Runx1, Gata3, Stat5, Il5, and Il13 found through CHIP-Seq [99]. In conclusion, PPAR-γ is crucial for the late stage Th2 effector functions performed by tissue-migrated Th2 cells by strongly regulating its epigenetic landscape and lipid metabolism. However, further studies are required to completely understand its mechanism involving Th2 functions.

**Figure 3:**
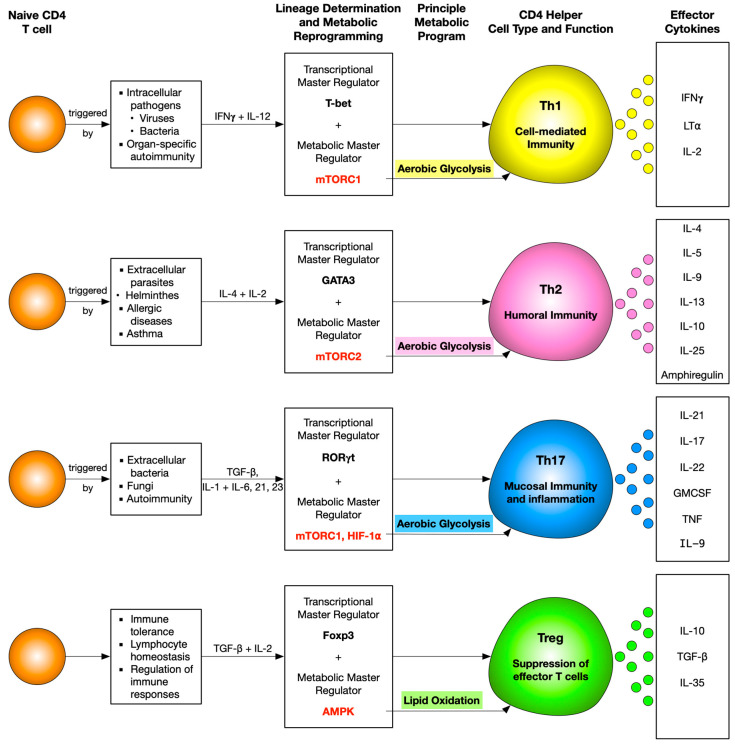
Snapshot of coordinated interplay between T cell differentiation and principle metabolic pathways monitoring different CD4 helper T cells. A naive CD4 T cell differentiates into different helper T cell subsets programmed by a master transcriptional regulator and predominant metabolic signaling molecule and pathway. Mammalian target of rapamycin complex 1 (mTORC1) is essential for Th1, mTORC2 for Th2, mTORC1 and HIF-1α for Th17, and AMP-activated protein kinase (AMPK) for Treg differentiation. Despite the different molecular metabolic regulators, Th1, Th2, and Th17 adopt aerobic glycolysis as their principle pathway, and on the other hand, Tregs adopt lipid oxidation.

Extracellular metabolites, namely ATP [100,101,102,103], short chain fatty acids (SCFAs) [104,105,106,107], glutamine [108], and an enzyme involved in tryptophan metabolism, indoamine 2,3-dioxygenase (IDO) [109,110], have also been reported to influence Th2 differentiation and functions. ATP and IDO potentiate, whereas SCFAs and glutamine suppress, Th2 differentiation and functions. However, a study showed that SCFAs may also enhance Th2-associated pathologies [104]. The metabolic landscape of all CD4 T-cell subsets is depicted in Figure 3.

### 3.3. Metabolic Regulation of Th17 Cells

Th17 cells have garnered significant attention due to their role in the pathology of several autoimmune diseases such as multiple sclerosis, rheumatoid arthritis, psoriasis, Crohn’s disease, and ulcerative colitis [111]. As many of these diseases lack effective therapies, understanding the metabolic control of Th17 cell development and function may manifest effective approaches to selectively impact Th17 cells, thus opening novel avenues for therapeutic manipulation.

IL-6 produced by innate immune cells, along with TGFβ, promotes the differentiation of naive T cells into Th17 cells [112,113,114]. IL-6 and TGFβ induce the expression of the key Th17 transcription factor RORγt via the STAT3 pathway [115]. IL-6 and TGFβ also promote the secretion of IL-21, which functions in an autocrine manner and increases RORγt expression, thus promoting IL-17 production. In addition to IL-17, Th17 cells mainly secrete proinflammatory cytokines including IL-22, GM-CSF, TNF, IL-9, and IL-21 [116]. IL-17 expression has been observed in lesions of patients with multiple sclerosis [117], and IL-17 and IL-22 have been found to promote blood–brain barrier disruption in the context of neuroinflammation [118].

The PI3K/AKT/mTOR pathway is a central regulator of cell metabolism. Utilizing genetic mouse models, several studies have found a selective role of mTORC1 (but not mTORC2) in Th17 differentiation [119,120]. Moreover, upregulation of mTORC1 but not mTORC2 has been observed in human Th17-mediated autoimmune diseases [121]. The AMP-activated protein kinase (AMPK), suppresses mTOR signaling by phosphorylating the TSC1/2 complexes, negative regulators of mTORC1. Higher mTOR activity resulting from impaired AMPK signaling either by deletion of AMPK regulator LKB1 [25] or AMPK target TSC-1 predisposes naive CD4 T cells to differentiate into Th17 cells [122]. On the other hand, poorer mTOR signaling due to AMPK activation impairs Th17 differentiation [123,124]. Thus, the PI3K–AKT–mTORC1 pathway and LKB1–AMPK pathway may serve as connecting pathways between environmental cues and T cell commitment to Th17 cells.

Th17 are characterized by inherent plasticity, and several recent studies have highlighted the metabolic control of plasticity in these cells. Using a mouse model of autoimmune disease, a recent study demonstrated that Th17 cells were functionally and metabolically heterogeneous [125]. It was shown that the Th17 subset with stemness-associated features had lower anabolic metabolic activity, whereas the subset that supported trans-differentiation into Th1-like cells displayed higher metabolic activity. Th17 cells with disrupted mTORC1 signaling acquired stemness-associated features and failed to induce autoimmune neuroinflammation or to develop into Th1-like cells. Thus, mTORC1 signaling serves as a central regulator of such Th17-cell fate decisions.

HIF-1α has also been shown to be essential for Th17 cells. HIF-1α levels are higher in Th17 cells than other T cell subsets such as Th1, Th2, and Tregs [126,127]. HIF-1α−/− T cells show impaired Th17 development and concomitantly higher Treg generation. HIF-1α may drive Th17 differentiation while simultaneously suppressing Treg induction by maintaining glycolytic activity in activated T cells [126]. Deletion of HIF-1α in Th17 cells leads to delayed development of experimental autoimmune encephalomyelitis (EAE) in a Th17-polarized transfer model of EAE [126], emphasizing the importance of HIF-1α in the pathogenicity of Th17 cells.

In addition to increasing glucose metabolism, T cells also promote lipid metabolism during their metabolic reprogramming upon activation. Unlike Tregs, which utilize exogenous fatty acids, Th17 cells primarily depend on de novo fatty acid synthesis (FAS) [128]. Acetyl-CoA carboxylase 1 (ACC1) is crucial for de novo fatty acid synthesis, and its inhibition impairs the generation of human and mouse Th17 cells but favors the development of Treg cells. T-cell-specific deletion of ACC1 in mice or in vivo treatment with an ACC-specific inhibitor has been shown to attenuate Th17 cell-mediated autoimmune disease [128]. The distinct metabolic requirements of Th17 cells and Treg cells regarding their dependency on ACC1-mediated de novo fatty acid synthesis may be exploited as a novel strategy to attenuate immune pathology.

When cells synthesize lipids from glucose, the final glycolytic product pyruvate is transferred into the mitochondria to form acetyl-CoA and then citrate, which then moves into the cytosol and gets converted to acetyl-CoA, which subsequently fuels FAS. Cytosolic acetyl-CoA can also be catalyzed in the mevalonate–cholesterol synthetic pathway. Inhibition of 3-hydroxy-3-methylglutaryl CoA reductase (HMGCR), a rate-limiting enzyme in the mevalonate–cholesterol pathway has been reported to impair Th17 differentiation [129,130]. Cholesterol precursor, desmosterol, and cholesterol derivatives, oxysterols, promote Th17 differentiation and function. Desmosterol selectively impacts the differentiation of Th17 cells without affecting Th1 and Treg differentiation [131]. Oxysterols (7β,27–OHC), derivatives of cholesterol, directly bind to RORγt ligand binding domain and enhance Th17 differentiation [132]. Thus, the lipogenic pathway plays a crucial role in regulating Th17 differentiation. Lipid metabolism is also essential for Th17 cell pathogenicity. CD5 antigen-like (CD5L) protein, a member of the scavenger receptor cysteine-rich superfamily, inhibits fatty acid synthase and its deletion converts non-pathogenic Th17 cells into pathogenic ones [133]. Collectively, Th17 differentiation and pathogenicity is tightly regulated by metabolic processes.

### 3.4. Metabolic Regulation of Treg Cells

Regulatory T cells (Tregs) are critical for maintaining peripheral tolerance and include natural Treg (nTreg) cells, which develop in the thymus, and induced Treg (iTreg) cells, which arise through the conversion of peripheral naive CD4 T cells. Tregs are crucial for preventing autoimmune diseases and limiting chronic inflammatory diseases. However, they also limit beneficial responses by suppressing effector T cells and limiting anti-tumor immunity.

Treg cells employ multiple mechanisms to mediate these suppressive effects [134,135,136]. These include suppression by inhibitory cytokines (IL-10, TGFβ, and IL-35), suppression by cytolysis via granzyme A, granzyme B, and perforin, and cytokine-deprivation-mediated apoptosis caused by the rapid consumption of IL-2. Tregs are also known to modulate dendritic cell (DC) maturation and function. Tregs downregulate the expression of CD80 and CD86 on DCs through trogocytosis, as well as stimulate DCs to express the enzyme indoleamine 2,3-dioxygenase (IDO), which catalyzes the conversion of tryptophan to kynurenine, which is toxic to T cells. Moreover, lymphocyte-activation gene 3 (LAG3) binding to MHC class II molecules also inhibits DC maturation and function. Tregs accumulating at the tumor site have significantly higher levels of inhibitory receptors, PD-1, CTLA-4, and TIM-3, as well as of CD39, an enzyme that participates in the conversion of adenosine-5′-triphosphate (ATP) to immunosuppressive adenosine (ADO). The tumor environment induces changes in the receptor profile of Treg cells, leading to Treg activation and upregulation of their suppressor activity.

Effector T cells (Teff) and regulatory T cells (Treg) require distinct metabolic programs to support their functions. Th1, Th2, and Th17 cells express high levels of Glut1 and are highly glycolytic. Tregs, in contrast, express low levels of Glut1 and are less dependent on glycolysis than Th1, Th2, and Th17 cells and primarily rely on FAO [64].

Signaling pathways such as phosphatidylinositol 3-kinase (PI3K), mitogen-activated protein kinase (MAPK), and mammalian target of rapamycin (mTOR) promote glycolysis in Treg cells [137,138]. Glucose uptake in Treg is mainly through the expression of Glut1. Interestingly, Glut1 enhances the proliferation of Treg cells but impairs their suppressive function [137]. Several studies have demonstrated that unrestrained glycolysis reduces Treg cell stability [126,139,140]. Blocking glycolysis has been shown to promote Treg cell generation. Moreover, amino acids are essential for effector T cells; however, iTreg cells seem to be less dependent on amino acids. For example, activation of naive CD4 T cells under conditions of glutamine deprivation resulted in their differentiation towards the Treg cell phenotype [67,141].

Foxp3, the key transcription factor of Treg cells, which is indispensable for their development, stability, and function, has also been shown to play a major role in regulating the metabolism of Treg cells. It was found that Glut1 expression is lowered by Foxp3 expression [137]. Foxp3 increases the expression of ETC protein complexes that can influence Treg function. Additionally, Foxp3 may decrease glycolysis by suppressing c-Myc expression by binding to the TATA box of the Myc gene [142]. Moreover, murine Tregs overexpressing a transgenic Glut1 had lower Foxp3 and CD25 expression and were incapable of suppressing colitis in an adoptive transfer model [137]. Deletion of HIF-1α, a transcription factor that promotes glycolysis, has been shown to increase Foxp3 expression [126]. In addition, Enolase-1, a glycolytic enzyme, binds to the Foxp3 promoter and its CNS2 region, thereby repressing the transcription of a splice isoform containing Exon-2 (FOXP3-E2), which is essential for the suppressive function of Tregs [143].

Several studies have pointed out the metabolic adaptations that allow Tregs to function in low-glucose, high-lactate environments. While such metabolic adaptations are essential for peripheral immune tolerance in ischemic tissues, they also weaken anti-cancer immune responses in the tumor microenvironment. Inflammatory sites are characterized by high lactate and low glucose levels, and such conditions strongly suppress effector T cells [144,145,146]. However, high concentrations of l-lactate and reduced glucose availability does not affect the proliferation or suppressive ability of Tregs [142]. This metabolic advantage of Tregs in high lactate environments is attributed to resistance to NAD+ depletion. Effector T cells depend on aerobic glycolysis and reduce NAD+ to NADH during the breakdown of glucose to pyruvate. Lactate dehydrogenase then catalyzes the reduction of pyruvate into lactate. Within high-lactate environments, lactate dehydrogenase (LDH) favors the reverse reaction and converts lactate into pyruvate while using NAD+ as a cofactor. Therefore, effector T cells face a redox imbalance during NAD+ insufficiency and glycolysis cannot happen. However, in Treg cells, Foxp3 inhibits glycolysis and promotes OXPHOS, thus allowing the Treg cells to generate NAD+ by oxidation in the TCA cycle [142]. Treg cells also show high resistance to amino acid deprivation, and depriving CD4 T cells of glutamine during activation leads to the generation of Treg cells, even in the presence of Th1-polarizing cytokines. Moreover, impaired glutamine uptake leads to decreased effector T-cell differentiation but does not affect Treg cell generation [68,147]. Thus, Treg cell metabolism offers them a survival advantage in nutrient-depleted environments.

The knowledge of the metabolic landscape essential for Treg proliferation and their suppression ability is crucial for their therapeutic manipulation in the future. In cancer, suppression of effector T cells by Tregs is detrimental, and rescuing this suppression is highly desirable. In contrast, suppression of immune responses by Tregs is essential during autoimmune diseases. Therefore, further research is required to understand how metabolic pathways can be targeted to induce or impair Treg-mediated suppression in particular diseases, and possible therapeutic interventions can be performed.

## 4. Targeting Metabolism for Cancer Immunotherapy

**Figure 4:**
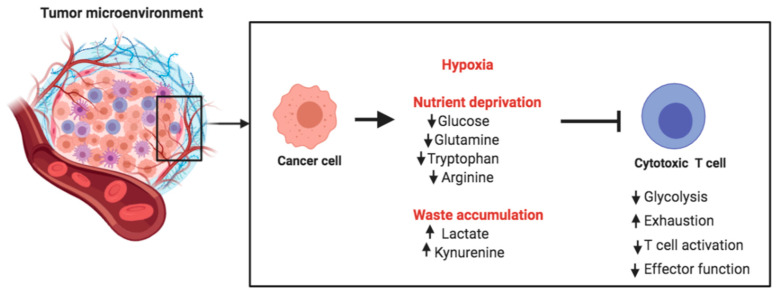
Metabolic challenges encountered by T cells in the tumor microenvironment. Cancer cells compete with tumor-infiltrating T cells for key nutrients that are also required for optimal T cell function. Tumor cells utilize aerobic glycolysis for their energetic requirements, thereby producing lactate as a byproduct in the extracellular milieu and acidifying the tumor microenvironment (TME). Thus, tumor-infiltrating T cells face multiple metabolic challenges in the hypoxic, acidic, and nutrient-deprived TME.

Cancer cells display remarkably high glucose and amino acid consumption. This causes nutrient deprivation in the tumor microenvironment (TME), thus posing metabolic challenges for tumor-infiltrating T cells. Glycolysis is essential for the differentiation of naive T cells into effector T cells. Thus, the glucose-deprived TME inhibits the differentiation and expansion of T cells. Cancer cells use high quantities of glutamine and secrete arginase, so that T cells in the TME also face competition for glutamine and arginine, both important nutrients for T cells (Figure 4).

Since glutamine is important for cancer cells, it has been an attractive therapeutic target for decades, but prior attempts to block glutamine metabolism in cancer patients have resulted in unacceptable toxicity [148]. A problem with using metabolic inhibitors in cancer therapy is that most metabolic pathways are not purely confined to cancer cells. Therefore, they can have toxic effects on non-cancerous cells. In a recent study, Leone et al. designed a prodrug form of the glutamine antagonist 6-diazo-5-oxo-L-norleucine (DON) known as JHU083 [149]. It circulates intact and inert but gets activated in the TME upon cleavage by cathepsins and other enzymes enriched in the TME. JHU083 not only impaired the glutamine metabolism pathway but also impaired glucose uptake by cancer cells, blocking the cancer cell metabolism, thereby increasing the nutrient and oxygen availability and lowering acidification in the TME. These metabolic changes in the TME effectively enhanced T cell survival and function required for the killing of tumor cells [149,150].

Several studies have also explored the possibility of targeting glucose metabolism for therapeutic purposes. For example, a recent study showed the effect of genetic ablation or pharmacological inhibition of PIM kinases in T cells [151]. This led to a reduction in glucose uptake and glycolytic activity in the T cells. Interestingly, the efficacy of the antitumor T cell response was significantly enhanced by inhibiting PIM kinases in tumor-bearing mice receiving adoptive T cell therapy. In addition, combination therapy using anti-PD1 + PIM inhibitor + adoptive transfer of T cells worked better for controlling tumor growth than monotherapy [151].

Apart from competing for key nutrients, cancer cells produce waste byproducts such as lactate and tryptophan metabolites (Figure 4). These byproducts are secreted into the TME, and their accumulation creates a metabolically toxic environment. Accumulation of lactate acidifies the microenvironment and can suppress T cell expansion, cytokine production, and cytotoxic activity. In vivo administration of esomeprazole, a proton pump inhibitor, has been used to buffer low pH at the tumor site in tumor-bearing mice. This was shown to improve the efficacy of tumor-infiltrating lymphocytes and delayed the progression of cancer [145]. This is an excellent example of targeting the acidic TME for rescuing anti-tumor immunity and controlling cancer progression.

Mitochondrial metabolism has also been targeted to test its therapeutic potential in cancer. The monocarboxylate transporter family includes MCT1, MCT2, MCT3, and MCT4 (encoded by SLC16A1, SLC16A7, SLC16A8, and SLC16A3 genes, respectively). These are transmembrane proteins essential for the bidirectional transport of lactate and other metabolites such as pyruvate and ketones, in and out of the cells [152]. MCT1 and MCT2 are not only required for lowering intracellular acidification by removing the excess lactate produced due to increased glycolysis in cancer cells but also for uptake of lactate and ketone bodies by the cells, which can feed the mitochondrial metabolism pathway in cancer stem cells [153]. Inhibitors of MCT1 have been shown to have promising outcomes in tumor models. Treatment with MCT1/2 inhibitor blocked the uptake of L-lactate and ketone bodies in cancer stem cells, which is required for anchorage-independent growth, proliferation, and survival [153]. Thus, targeting mitochondrial metabolism by MCT inhibitors may have important clinical implications for the eradication of cancer stem cells. A recent study also demonstrated that lipid metabolism in melanoma cells leads to higher antigen presentation, which increases melanoma sensitivity to T-cell-mediated killing, thereby providing another avenue for harnessing metabolism for therapeutic interventions [154].

Targeting amino acid metabolism has proven to be another attractive strategy for cancer immunotherapy. Indoleamine 2,3-dioxygenase 1 (IDO1) is an enzyme involved in tryptophan metabolism. It catalyzes an important step in the oxidation of L-tryptophan into kynurenine. IDO1 is expressed by many tumors, and its high expression has been associated with tumor progression and shortened patient survival [155]. IDO1 mediates T cell suppression by depleting tryptophan in the TME and by increasing kynurenine, which suppresses effector T cell function and also activates Tregs. IDO1 inhibitors have been shown to rescue these effects, and the combination of IDO1 inhibitors with the PD1 checkpoint blockade has resulted in better outcomes in clinical trials [156].

Antitumor responses of CD8 T cells can also be enhanced by targeting cholesterol metabolism. Acyl-CoA acyltransferase 1 (ACAT1) is an important cholesterol esterification enzyme, and its inhibition blocks cholesterol esterification in T cells. This causes an increase in the amount of cholesterol in the plasma membrane of CD8 T cells, leading to efficient TCR clustering and immunological synapse formation [157]. Therefore, the ACAT1 inhibitor avasimibe enhances the proliferation and effector function of CD8 T cells and ACAT1-deficient CD8 T cells performed better in controlling tumor growth [157]. Collectively, these studies and many others highlight the importance of understanding T cell metabolism as well as cancer metabolism in order to successfully target metabolic pathways for cancer immunotherapy.

## 5. Concluding Remarks

It is clear that T cell function and differentiation are intricately linked with metabolism, thereby providing an opportunity of manipulating T cell function and fate by blocking or potentiating specific metabolic pathways. However, in order to do so, we require a complete understanding of all the metabolic pathways that are engaged by T cells during different stages of their lifespan, from their development in the thymus to their activation and differentiation in the periphery, until they die by apoptosis.

Several studies have provided evidence for manipulating T cell differentiation and function by specifically targeting regulators of cellular metabolism. One strategy is to shift the balance between different CD4 T cell subsets. An impaired balance between Th17 and Treg cells is involved in various autoimmune diseases. Th17 cells contribute to inducing inflammation, whereas Treg cells restrain inflammation and are crucial for the maintenance of immune tolerance. Modulating metabolism using specific small molecular compounds could be a potential way of shifting the Th17/Treg cell balance and, therefore, has a promising therapeutic role. Rapamycin is an immunosuppressive drug that is used clinically to treat autoimmunity and graft rejection. It is shown to inhibit Th17 cell differentiation but promotes Treg cell generation [158,159]. A recent study found that rapamycin regulates the balance between Th17 and Treg cells by modulating cellular metabolism [160]. Th17 cells primarily depend on glycolysis activity, whereas Treg cells rely on fatty acid oxidation (FAO). Rapamycin blocks glycolysis by inhibiting glucose uptake and the expression of hexokinase 2 in induced Th17 cells but promotes FAO in induced Treg cells, thereby contributing to a shift in the Treg/Th17 balance. Furthermore, Th17 cells have a higher expression of pyruvate dehydrogenase kinase 1 (PDK1) than other CD4 T cell subsets. PDK1 is known to promote aerobic glycolysis through the inhibition of pyruvate dehydrogenase. Gerriets and coworkers showed that inhibition of PDK1 using dichloroacetate selectively impairs Th17 proliferation and survival, thereby reducing T-cell-mediated inflammation in inflammatory bowel disease (IBD) and experimental autoimmune encephalitis (EAE) disease models [161].

The use of metabolic modulators has also been tested in the autoimmune disease systemic lupus erythematosus (SLE). CD4 T cells from SLE patients have elevated glycolysis and mitochondrial oxidative metabolism. Interestingly, treatment with 2-deoxy-d-glucose (glycolysis inhibitor) and metformin (mitochondrial electron transport chain complex I inhibitor) normalized CD4 T cell metabolism and reversed disease phenotypes of SLE in animal models as well as in cells from SLE patients [162]. Other promising metabolic regulators that improved SLE disease outcomes include rapamycin [163] and *N*-acetylcysteine [164,165], which block the mTOR pathway.

Targeting cellular metabolism could provide an opportunity to manipulate T cell function for cancer immunotherapy. For example, treatment with fenofibrate, a PPARα agonist that is known to enhance FAO in T cells, leads to improved function of CD8 T cells cultured in hypoglycemic and hypoxic conditions, and of tumor infiltrating lymphocytes from tumor-bearing mice treated with fenofibrate and transplanted to secondary recipients [166]. This holds great clinical potential as fenofibrate treatment was shown to synergize with anti-PD1 therapy [166], indicating that targeting metabolism along with checkpoint blockade can be a promising therapeutic approach.

In the context of cancer, immunosuppression in the tumor microenvironment is at least in part driven by the inability of T cells to acquire the nutrients to support their metabolism. Tumor cells have a high consumption of glucose and glutamine, which may cause nutrient deprivation for effector T cells. Thus, T cells infiltrating the tumor microenvironment experience metabolic stress, causing anergy or T cell dysfunction [167]. A thorough understanding of cellular metabolism of T cells will allow us to target metabolic pathways in order for T cells to function within the tumor microenvironment. In adoptive T cell immunotherapy, T cells from a patient are genetically manipulated and expanded in vitro and then transferred back into the patient. These cells need to proliferate and perform effector functions to clear tumors. Therefore, further research on manipulating T cell metabolism to enhance cell longevity and function is essential for adoptive immunotherapies.
